# HRCT diagnosis of combined pulmonary fibrosis and emphysema in a patient of chronic obstructive pulmonary disease with pulmonary hypertension and clinical or radiograph suspicion of pulmonary fibrosis

**DOI:** 10.1259/bjrcr.20150070

**Published:** 2016-11-02

**Authors:** Kataveeranahally Shekar Manjunath, Hirennappa Udnur

**Affiliations:** ^1^Department of Radiology , Columbia Asia Hospital, Bangalore, India; ^2^Department of Pulmonary Medicine, Columbia Asia Hospital, Bangalore, India

## Abstract

Combined pulmonary fibrosis and emphysema (CPFE) is a unique pulmonary condition characterized by simultaneous coexistence of both upper lobe emphysema and lower lobe fibrosis. Pulmonologists should be aware of the entity while evaluating patients with chronic obstructive pulmonary disease (COPD) or pulmonary fibrosis. Airflow and lung volume are relatively preserved but oxygenation is disproportionately impaired in patients with CPFE. We describe a case of an 83-year-old male patient with past history of heavy smoking, in whom the search for the cause of pulmonary arterial hypertension and exercise-induced arterial oxygen desaturation disproportionate to be explained by COPD resulted in a diagnosis of CPFE. He complained of dyspnoea on exertion and non-productive cough. Physical examination revealed basal Velcro rales and clubbing. Chest radiography showed prominent vascular markings, preserved lung volume and subtle fibrosis of the bases. Definitive diagnosis was made on CT scan of the chest, which revealed upper lobe emphysema and lower lobe fibrosis and honeycombing. The patient was managed by long-term oxygen therapy, inhaled corticosteroid, long-acting bronchodilator and antimuscarinic agents, diuretic, pirfenidone (antifibrotic agent), proton pump inhibitor and *N*-acetyl cysteine (antioxidant). We emphasize the importance of the diagnosis of CPFE in early stages through CT in a case of COPD with clinical, laboratory and chest radiographic evidence of fibrosis and the fact that CPFE is associated with pulmonary hypertension, a poor prognostic indicator.

## Clinical findings

An 83-year-old male with a past history of smoking and chronic obstructive pulmonary disease (COPD) presented with persistent non-productive cough, exertional dyspnoea and wheezing that was relieved by inhaler and right-sided pleurisy for 2 days. There were no symptoms of obstructive sleep apnoea. Epworth Sleepiness Scale for obstructive sleep apnoea was performed and score was < 8. There were no symptoms or signs suggestive of connective tissue disorder. Clinical examination revealed bilateral pedal oedema and bilateral basal Velcro rales. SpO_2_ was 93% at rest and 88% after exercise. Echocardiogram revealed normal chamber dimensions, no regional wall motion abnormality, moderate tricuspid regurgitation, pulmonary arterial systolic pressure of 60 mmHg, normal left ventricular function (left ventricular ejection fraction: 60%) and Grade 1 left ventricular diastolic dysfunction. The patient did not cooperate for pulmonary function test (PFT). Screening tests for pulmonary thromboembolism (colour Doppler of lower limbs and D-dimer assay) were negative. Screening chest X-ray showed prominent vascular markings in the upper and mid zones with subtle fibrosis in the lower zones. A suspicion of fibrotic process in the lungs was raised and further evaluation by high-resolution CT (HRCT) was ordered.

## Imaging findings—high-resolution CT chest

**Figure 1.**
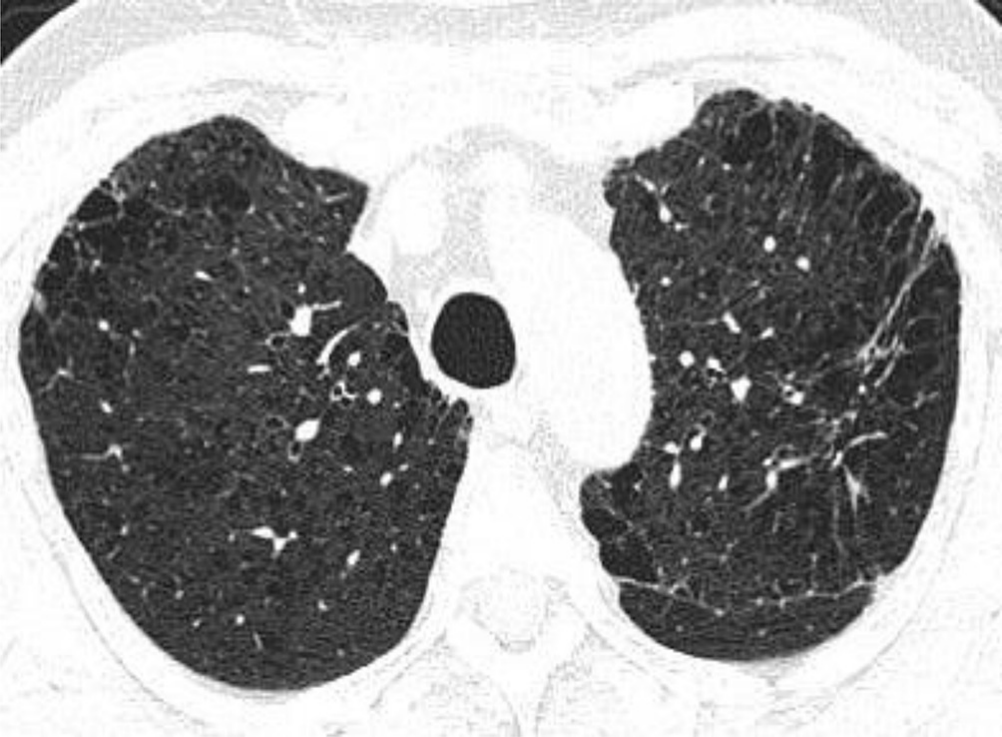
Centrilobular and paraseptal emphysematous changes in bilateral upper lobes and apical segment of left lower lobe. Also noted is a fibrotic lesion in the apicoposterior segment of the left upper lobe.

**Figure 2.**
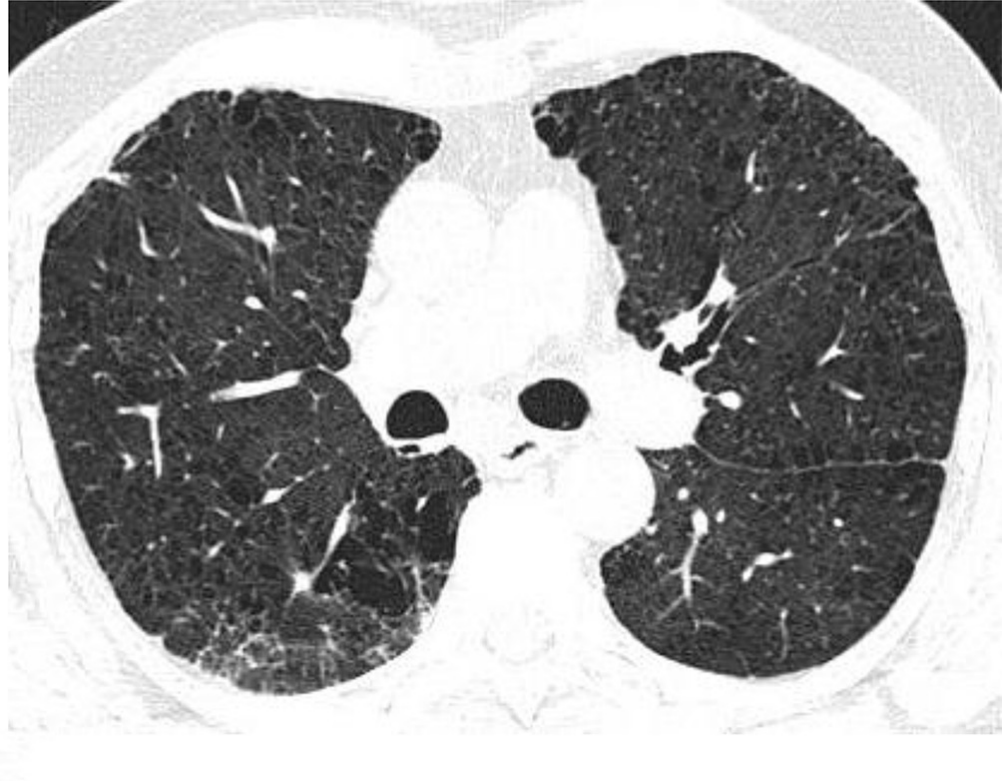
Centrilobular and paraseptal emphysematous changes in bilateral upper lobes and apical segments of bilateral lower lobes. Also noted is subtle ground-glass opacity in the apical segment of the right lower lobe.

**Figure 3.**
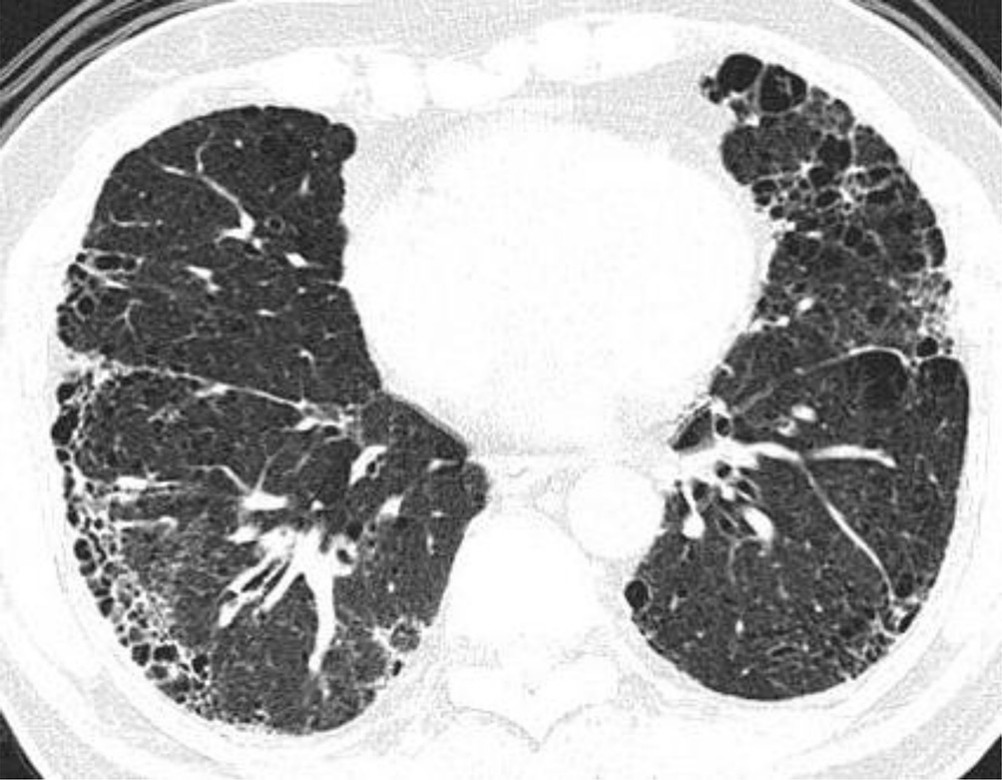
Reticular opacities, interlobular interstitial thickening with honeycombing, ground-glass opacity and emphysematous changes in bilateral lower lobes, right middle lobe and lingular segment of the left upper lobe.

**Figure 4.**
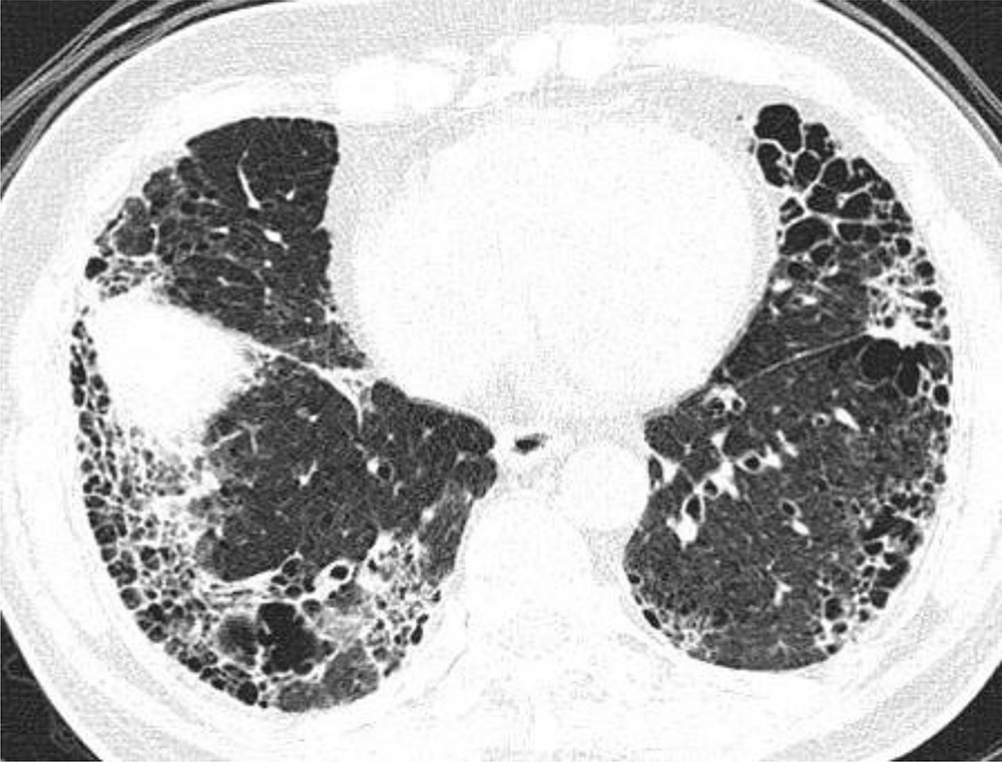
Reticular opacities, interlobular interstitial thickening with honeycombing and ground-glass opacity are more prominent in the lower lobes in subpleural locations.

Extensive centrilobular and paraseptal emphysema were seen in bilateral lungs with upper lobe predominance (Figures 1 and 2). Reticular opacities with honeycombing, traction bronchiectasis and subtle ground-glass opacity were seen in bilateral lungs with subpleural and lower lobe predominance (Figures 3 and 4). There was no significant lymphadenopathy or pleural effusion. Pulmonary artery and cardiac chamber dimensions were normal.

## Final diagnosis and treatment

Based on HRCT findings, the patient was diagnosed as a case of combined pulmonary fibrosis and emphysema (CPFE) and managed by long-term oxygen therapy, oral theophylline, inhaled corticosteroid, long-acting bronchodilator and antimuscarinic agents, diuretic, pirfenidone (antifibrotic agent), proton pump inhibitor and *N*-acetyl cysteine (antioxidant). The patient was advised for regular follow-up.

## Discussion

Unanimous definition of CPFE is yet to be adopted. Broadly, it includes all patients with simultaneous coexistence of pulmonary fibrosis and emphysema.

CPFE as a unique pulmonary condition characterized by the simultaneous coexistence of lower lobe fibrosis and upper lobe emphysema was first described by Wiggins et al^1^ in 1990. But Cottin et al^2^ were the first to comprehensively describe the syndrome resulting from CPFE in 2005. CPFE was recognized in 8% cases of idiopathic pulmonary fibrosis (IPF) in a study by Ryerson et al.^3^

Most patients with CPFE are elderly males around age 65 years or older with a history of cigarette smoking, the most commonly cited environmental risk factor.^2^ Agrochemical compounds are also attributed as possible risk factors.^4^ CPFE patients classically present with progressive shortness of breath, with an average symptom duration of about 3 years before diagnosis.^2^ As in any fibrotic interstitial lung disease, bilateral basal Velcro rales and clubbing are the main findings on clinical examination.^2^

It has been reported that CPFE patients are often misdiagnosed as cases of COPD and treated with short-acting bronchodilators and long-acting anticholinergics.^3^ Diagnosis of CPFE is confirmed essentially on the basis of findings on HRCT. Clinical data and pathological findings are helpful in some cases.

The three main clinical scenarios in which CPFE should be considered are: (i) patients with PFTs showing normal spirometry, or mild obstructive or restrictive pattern and normal lung volumes but with severely diminished diffusing capacity of the lung for carbon monoxide (DLCO).^2,5^ Reason for this pattern of abnormality in PFT and DLCO is that volume loss and low compliance owing to fibrosis is compensated by hyperinflation and high compliance due to emphysema but pulmonary emphysema and fibrosis have additive effects on carbon monoxide transfer and exercise hypoxemia.^2,5^ But this pattern is not specific for CPFE and is also seen in pulmonary vascular diseases, interstitial lung diseases and emphysema.^6^ (ii) Patients with pulmonary hypertension and abnormal PFTs, especially with a mixed restrictive/obstructive pattern.^2,7^ (iii) Patients with normal or near-normal spirometry but with hypoxemia at rest or upon exertion and oxygen requirement disproportionate to spirometric abnormality.^2,5^

The exact pathogenesis of CPFE is unknown. Cigarette smoking is quoted as the possible aetiology in majority of the reported cases. Exposure to cigarette smoke led to synchronous development of pulmonary fibrosis and emphysema in the canine model.^8^

Lower lobe interstitial fibrotic changes and upper lobe emphysema are the typical radiologic findings in the CPFE syndrome. Paraseptal, centrilobular emphysematous and bullous changes are seen in CPFE.^2,9^ Interstitial fibrotic changes include honeycombing and reticular abnormalities. Ground-glass attenuation areas are also commonly present.^2^ Sometimes, ground-glass attenuation is the sole abnormality suggesting interstitial lung disease and biopsy is required in this setting^10^ to differentiate CPFE from other smoking-related lung diseases.

Specific treatment does not exist currently for CPFE syndrome. The present guidelines are based on evidence related to the underlying conditions of chronic obstructive lung disease and IPF.^11^ Pulmonary hypertension, acute lung injury and lung cancer are listed as complications of CPFE.

Pulmonary hypertension is said to occur more commonly and severely in patients with CPFE than in those with isolated IPF.^12^ It does not significantly respond to medical therapy.^13^ The patients are managed with oxygen therapy and lung transplantation.

Mortality is significant in patients with CPFE. The 1-year survival is as low as 60% in patients with confirmed pulmonary hypertension.^13^ In their study, Ryerson et al^3^ concluded that mortality rate of patients with CPFE is similar to those with isolated IPF, although patients with CPFE had more extensive smoking history, greater oxygen requirements, higher pulmonary artery pressure and poorer gas exchange.^3^ Similar mortality in IPF as compared twith CPFE was attributed to greater amount of fibrosis in IPF compensating for other mortality increasing factors in CPFE.^3^

## Conclusions

We emphasize the importance of suspecting the diagnosis of CPFE in a patient with COPD with evidence of fibrotic process in the lungs on clinical examination or chest radiographic findings with echocardiographic evidence of pulmonary hypertension. We also emphasize that CPFE is a distinct pulmonary condition that, in a classic case, can be definitely diagnosed by HRCT.

## Learning points

1. CPFE is a unique pulmonary condition characterized by the simultaneous occurrence of both upper lobe emphysema and lower lobe pulmonary fibrosis.
2. CPFE is seen mostly in elderly males with significant smoking history.
3. CPFE has a progressive course, with an average duration of symptoms of about 3 years before diagnosis.
4. CPFE patients are often misdiagnosed as cases of COPD and treated accordingly.
5. HRCT is the modality of choice, and it typically demonstrates the characteristic upper lobe emphysema with lower lobe interstitial fibrotic changes.
6. With the classic clinical and HRCT findings, a confident diagnosis can be made without the need for a surgical lung biopsy.
7. Unfortunately, CPFE has no effective treatment options available other than possible lung transplant and overall has a poor prognosis.
8. The prevalence of pulmonary arterial hypertension is high in CPFE, and its presence at diagnosis is by itself a poor prognostic sign.
9. Along with pulmonary arterial hypertension, lung cancer and acute lung injury are listed as complications. Close follow-up is essential.
10. Mortality rate in patients with CPFE is similar to that in patients with isolated IPF.

## Consent

Informed consent was obtained to publish this case (including images and data) and is held on record.

## References

[1] Wiggins J, Strickland B, Turner-Warwick M Combined cryptogenic fibrosing alveolitis and emphysema: the value of high resolution computed tomography in assessment. Respir Med 1990; 84: 365–9. 2247666 10.1016/s0954-6111(08)80070-4

[2] Cottin V, Nunes H, Brillet PY, Delaval P, Devouassoux G, Tillie-Leblond I et al Combined pulmonary fibrosis and emphysema: a distinct underrecognised entity. Eur Respir J 2005; 26: 586–93. 16204587 10.1183/09031936.05.00021005

[3] Ryerson CJ, Hartman T, Elicker BM, Ley B, Lee JS, Abbritti M et al Clinical features and outcomes in combined pulmonary fibrosis and emphysema in idiopathic pulmonary fibrosis. Chest 2013; 144: 234–40. 23370641 10.1378/chest.12-2403

[4] Daniil Z, Koutsokera A, Gourgoulianis K Combined pulmonary fibrosis and emphysema in patients exposed to agrochemical compounds. Eur Respir J 2006; 27: 434. 10.1183/09031936.06.00124505 16452604

[5] Silva DR, Gazzana MB, Barreto SS, Knorst MM Idiopathic pulmonary fibrosis and emphysema in smokers. J Bras Pneumol 2008; 34: 779–86. 19009210 10.1590/s1806-37132008001000005

[6] Kiakouama L, Cottin V, Glerant JC, Bayle JY, Mornex JF, Cordier JF Conditions associated with severe carbon monoxide diffusion coefficient reduction. Respir Med 2011; 105: 1248–56. 21454061 10.1016/j.rmed.2011.03.004

[7] Simonneau G, Robbins IM, Beghetti M, Channick RN, Delcroix M, Denton CP et al Updated clinical classification of pulmonary hypertension. J Am Coll Cardiol 2009; 54: S43–54. 19555858 10.1016/j.jacc.2009.04.012

[8] Hammond EC, Auerbach O, Kirman D, Garfinkel L Effects of cigarette smoking on dogs. Arch Environ Health 1970; 21: 740–53. 5478559 10.1080/00039896.1970.10667328

[9] Kitaguchi Y, Fujimoto K, Hanaoka M, Kawakami S, Honda T, Kubo K Clinical characteristics of combined pulmonary fibrosis and emphysema. Respirology 2010; 15: 265–71. 20051048 10.1111/j.1440-1843.2009.01676.x

[10] Jankowich MD, Polsky M, Klein M, Rounds S Heterogeneity in combined pulmonary fibrosis and emphysema. Respiration 2008; 75: 411–17. 17684315 10.1159/000107048

[11] Raghu G, Collard HR, Egan JJ, Martinez FJ, Behr J, Brown KK et al An official ATS/ERS/JRS/ALAT statement: idiopathic pulmonary fibrosis: evidence-based guidelines for diagnosis and management. Am J Respir Crit Care Med 2011; 183: 788–824. 21471066 10.1164/rccm.2009-040GL PMC5450933

[12] Mejía M, Carrillo G, Rojas-Serrano J, Estrada A, Suárez T, Alonso D et al Idiopathic pulmonary fibrosis and emphysema: decreased survival associated with severe pulmonary arterial hypertension. Chest 2009; 136: 10–15. 19225068 10.1378/chest.08-2306

[13] Cottin V, Le Pavec J, Prévot G, Mal H, Humbert M, Simonneau G et al Pulmonary hypertension in patients with combined pulmonary fibrosis and emphysema syndrome. Eur Respir J 2010; 35: 105–11. 19643948 10.1183/09031936.00038709

